# Deep Learning-Assisted Porosity Assessment for Additive Manufacturing Components Using Ultrasonic Coda Waves

**DOI:** 10.3390/s26020478

**Published:** 2026-01-11

**Authors:** Xinyi Yuan, Xianmin Chen, Fang Wen

**Affiliations:** 1 School of Aerospace Engineering, Xiamen University, Xiamen 361005, China; yuanxinyi@xmu.edu.cn; 2 National Key Laboratory of Strength and Structural Integrity, Aircraft Strength Research Institute of China, Xi’an 710065, China; 3 School of Energy and Power Engineering, Beihang University, Beijing 100190, China; wenf@buaa.edu.cn; 4 AVIC China Aero-Polytechnology Establishment, Beijing 100028, China

**Keywords:** additive manufacturing (AM), porosity, ultrasonic coda wave, non-destructive evaluation (NDE), deep learning (DL)

## Abstract

**Highlights:**

An innovative approach for porosity assessment of Additive Manufacturing (AM) components is proposed, integrating Deep Learning (DL) with ultrasonic coda waves.
The integration of feature-rich ultrasonic coda waves with the strong feature extraction of DL enables highly accurate porosity evaluation for AM components.

**What are the main findings?**

- Ultrasonic coda waves can propagate fully inside the AM components and exhibit high sensitivity to changes in the AM components’ porosity.
- The introduction of a Multi-head Attention Mechanism (MAM) effectively improves the porosity assessment accuracy of the proposed coda-CNN-MAM network.

**What are the implications of the main findings?**

- Nondestructive Evaluation (NDE) of porosity in AM components is achieved using ultrasonic coda waves.
- The integration of coda waves with DL provides a highly accurate framework for assessing porosity in AM components.

**Abstract:**

The porosity of additive manufacturing components significantly impacts their mechanical properties, thereby limiting their widespread application in engineering. Current porosity assessment predominantly relies on destructive testing, underscoring the urgent need for accurate in situ non-destructive testing methods. In this paper, we propose a novel deep learning-assisted non-destructive testing method for porosity assessment in additive manufacturing components. Our approach leverages the high sensitivity of ultrasonic coda waves to minute internal material changes, combined with the powerful feature extraction capability of deep learning. Experimental results demonstrate that ultrasonic coda waves are sensitive to porosity variations in additive manufacturing components. Due to the porosity of additive manufacturing components involves multi-dimensional micro-structural features, conventional parameters such as the correlation coefficient and relative velocity change cannot establish an effective mapping relationship, despite their variation with porosity, thus precluding accurate inversion. To address this challenge, we propose a coda–convolutional neural network–multi-head attention mechanism network. Ultrasonic coda waves can fully interact with pores inside additive manufacturing components, and their signals are rich in porosity-related features. The introduction of deep learning significantly enhances the ability to extract such features. The trained network achieves high-precision porosity prediction with an accuracy of 98%. Our proposed approach reveals the complementary integration of ultrasonic coda waves and deep learning methods: the former provides high sensitivity to porosity changes, while the latter addresses the limitations of difficult extraction of relevant features and unclear complex mapping relationships. This collaborative framework establishes a new solution for high-precision non-destructive testing of additive manufacturing components.

## 1. Introduction

Additive Manufacturing (AM) technology, especially metal AM technology, such as Selective Laser Melting (SLM), has found increasingly widespread application in high-end manufacturing sectors, including aerospace, biomedicine, and automotive manufacturing. The core strengths of this technology lie in one-piece forming for complex geometries, high material utilization rates, and rapid customization capabilities [1,2]. However, the intense physical and chemical reactions during AM processing often lead to melt pool instability and powder splashing [3,4], inevitably introducing internal defects such as pores and incomplete fusion [5,6]. These tiny pores, which are prone to inducing stress concentration, will markedly degrade the fatigue strength, fracture toughness, and dynamic mechanical properties of AM components, directly endangering the service safety and operational reliability of the entire structure [7,8,9]. Therefore, accurate assessment of porosity in AM components is not only a technical prerequisite for guaranteeing the performance of AM components and meeting the demands of harsh service conditions, but also a pivotal enabler for advancing AM technology from a manufacturable stage to a reliable one.

Leveraging its nondestructive characteristic, high efficiency, and excellent adaptability to various materials and complex geometries, Ultrasonic Testing (UT) holds significant promise for porosity assessment in AM components [10,11,12,13]. Huang et al. [14] assessed the printing quality of 316L AM components based on the attenuation and wave velocity of ultrasonic waves. Specifically, Their experiments confirmed a distinct positive correlation of attenuation with porosity and a negative correlation of velocity with porosity. This finding provides a new method for the Non-Destructive Evaluation (NDE) of the print quality of AM components. Zhang et al. [15] also employed ultrasonic waves to assess the porosity of AM components. They established a relationship between porosity and both ultrasonic attenuation and wave velocity across different frequencies. The observed trends aligned with prior findings, offering valuable guidance for frequency selection in the UT of AM component porosity. Nevertheless, conventional ultrasonic testing techniques continue to face bottlenecks: the sizes of pores (typically on the micrometer scale) [5] typically present in AM components are frequently 1–2 orders of magnitude smaller than the half wavelength of the applied ultrasonic wave (typically on the millimeter scale) [14,15], which in turn directly constrains the accuracy and sensitivity of quantitative porosity assessment for AM components.

When ultrasonic waves propagate in heterogeneous media, they undergo multiple scattering, resulting in the generation of coda waves. Unlike direct waves, coda waves travel significantly longer paths and can fully interact with the internal structure of the assessed component, thus including a rich trove of information about the material’s microstructure and mechanical properties. Consequently, coda waves exhibit high sensitivity to subtle structural alterations, facilitating accurate assessments of mechanical performance [16,17,18]. Snider et al. [19] pioneered Coda Wave Interferometry (CWI), demonstrating its efficacy in detecting structural state changes, pattern recognition, and defect diagnosis. Building on this, this method has been widely adopted to assess subtle changes in components. Wuttke et al. [20] employed CWI to assess the wave velocity variations caused by small perturbations in soils, such as stress and void ratio, and their experimental results highlighted the significant potential of CWI for time-lapse monitoring of soils. In addition, Martino et al. [21] conducted an investigation into the impact of pore characteristics in volcanic rocks on ultrasonic wave propagation. Specifically, their findings revealed that pore size, shape, volume fraction, and spatial distribution exert a measurable influence on the amplitude and phase of coda waves. The aforementioned research demonstrates the high sensitivity of coda waves to pores, indicating their considerable potential for porosity assessment. However, the specific application of coda waves for quantifying porosity in AM components remains largely unexplored.

Conventionally, coda wave analysis relies on established algorithms such as the stretching method [22] and Moving Window Cross-Correlation (MWCC) [23]. While these techniques are widely used, the high sensitivity of coda waves to material properties also renders them susceptible to environmental noise. Consequently, when processed with such conventional methods, noise interference can significantly compromise assessment accuracy. Given its powerful feature extraction capabilities, Deep Learning (DL) presents a promising avenue to overcome this limitation [24,25,26]. Currently, there is a growing trend of integrating DL with UT to enhance detection accuracy. Wen et al. [27] integrated Electromagnetic Acoustic Resonance (EMAR) with a One-Dimensional Convolutional Neural Network (1D-CNN) to achieve accurate non-contact characterization of material hardness. This method demonstrated high predictive accuracy, with the introduced DL framework successfully overcoming the critical challenge of low Signal-to-Noise Ratio (SNR) in conventional EMAR detection. Shin et al. [28] developed an anomaly detection model for early damage identification in concrete structures affected by the Alkali-Silica Reaction (ASR) by integrating coda waves with DL, achieving high-precision detection. These studies collectively confirm DL’s powerful capability in extracting critical damage-related features from complex UT signals, thereby strongly supporting the feasibility of combining DL with coda waves for porosity assessment in AM components.

In this paper, we propose a novel method for assessing the porosity of AM components by integrating DL with the ultrasonic coda wave. Firstly, we investigate the interaction between ultrasound coda waves and porosity of AM components, using correlation coefficients and relative velocity changes to characterize the coda wave variations. The research results indicate that ultrasonic coda wave is indeed sensitive to changes in porosity within AM components. However, the porosity of AM components is characterized by multi-dimensional micro-structural features, and it is difficult to establish a linear mapping relationship with a single parameter, rendering high-precision inversion unachievable through conventional methods. To overcome this challenge, we propose a Coda-CNN-Multi-head Attention Mechanism (MAM) network, which combines the powerful feature extraction capability of DL with the characteristic of the coda wave containing multiple porosity-related features. By comparing separately with the assessment results of the CNN trained only on coda wave signals and the proposed network trained only on the primary wave signals, the significant effectiveness and prominent advantages of the proposed network in the porosity evaluation of AM components are verified. This work establishes a new framework for porosity assessment in AM components, demonstrating the potential of combining DL with ultrasonic coda wave in NDE.

## 2. Theoretical Considerations

**Figure 1:**
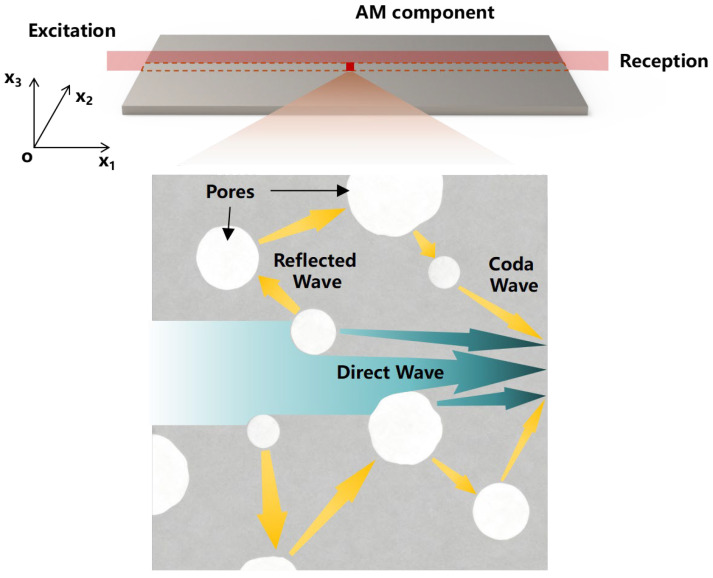
Schematic diagram of ultrasonic guided wave propagation and coda wave generation under the action of pores in AM components.

Ultrasonic guided waves offer several advantages for Non-Destructive Testing (NDT), including long propagation distances, high detection efficiency, sensitivity to internal defects, and the capability to inspect components with complex geometries. These characteristics collectively make them well-suited for the assessment of AM components. For an isotropic thin plate, as shown in Figure 1, a spatial Cartesian coordinate system is established. The governing partial differential equation for the displacement of any material point within the plate is given by [29]:

(1) $$\mu u_{i,jj}+\left(\lambda +\mu \right)u_{j,ji}+\rho f_{i}=\rho {\ddot{u}}_{i},$$

where *i* and *j* are taken as 1, 2, 3, respectively, and *f_i_* and *u_i_* represent the internal force and displacement component, respectively. *ρ* is the density of the material, and *λ* and *μ* are the Lamé constants. To separate the propagation characteristics of longitudinal (dilatational) and transverse (shear) waves, the displacement field is decomposed into a scalar potential ∇Φ (*x*_1_, *x*_3_) (governing longitudinal waves) and a vector potential ∇ × ψ (*x*_1_, *x*_3_) (governing transverse waves). Substituting the above potential decomposition into Equation (1), we derive the wave equations for longitudinal and transverse waves in the plate:

(2) $$\frac{\partial^{2}\Phi }{\partial x_{1}^{2}}+\frac{\partial^{2}\Phi }{\partial x_{3}^{2}}=\frac{1}{c_{L}^{2}}\cdot \frac{\partial^{2}\Phi }{\partial t^{2}},$$

(3) $$\frac{\partial^{2}\psi }{\partial x_{1}^{2}}+\frac{\partial^{2}\psi }{\partial x_{3}^{2}}=\frac{1}{c_{T}^{2}}\cdot \frac{\partial^{2}\psi }{\partial t^{2}},$$

where *c_L_* and *c_T_* represent the longitudinal and transverse wave velocities in the plate, respectively. The solutions to Equations (2) and (3) involve combinations of sine and cosine functions. By applying the free-stress boundary conditions at the plate’s upper and lower surfaces and using the separation of variables method, we obtain the characteristic equations for symmetric and anti-symmetric guided wave modes (Lamb wave modes) [29]:

(4) $$\frac{tan(qh)}{tan(ph)}+\frac{4k^{2}pq}{{\left(q^{2}-k^{2}\right)}^{2}}=0,$$

(5) $$\frac{tan(qh)}{tan(ph)}+\frac{{\left(q^{2}-k^{2}\right)}^{2}}{4k^{2}pq}=0,$$

where *h* is the plate thickness, *k* is the wavenumber, *p*^2^ = *ω*^2^/*c_L_*^2^ − *k*^2^, and *q*^2^ = *ω*^2^/*c_T_*^2^ − *k*^2^. The above equations allow for the calculation and analysis of guided wave propagation characteristics within the plate. As previously noted, AM components inherently contain pores. Li et al. [30] have demonstrated that ultrasonic propagation characteristics can be altered even by interactions with defects smaller than half the wavelength. Consequently, when ultrasonic waves travel through these AM components, the inherent pores inevitably modify the original ultrasonic wavefield and propagation characteristics, giving rise to coda waves. As illustrated in Figure 1, the propagation path of the coda wave is significantly longer than that of the primary wave. This extended path enables more comprehensive interaction with the internal structure of the component, thereby making the coda wave more sensitive to subtle internal changes within the component.

A currently prevalent technique for coda wave analysis is CWI. The principle of CWI can be described by the path superposition theory, which states that the wavefield at any given point can be represented as the superposition of waves propagating along all possible paths, as follows [31]:

(6) $$u(t)=\sum_{P}S_{P}(t),$$

where *u*(*t*) denotes the wavefield recorded at a specific location, while S*_p_*(*t*) represents the set of all possible wave propagation paths within the component. Thus, the wavefield can be understood as the superposition of waves that have traveled along all possible scattering paths. These paths give rise to the primary wave, which arrives first, followed by singly scattered waves, and finally the coda waves, which result from multiple scattering within the component. Consider a wave that has traveled a distance l along a path *P*. In the presence of a slight perturbation δl between the transmitter and receiver, the resultant variation in the wavefield is formulated as follows [31]:

(7) exp(ikδl)∼1+ikδl,

(8) $$\left|\delta u^{phase}\right|=\left|k\delta lu\right|.$$

According to Equation (8), a slight perturbation δl manifests as a phase shift in the received signals, directly translating to a change in their Time-of-Flight (TOF). This establishes the variation in time difference as a sensitive measure of minor perturbations within the component.

## 3. Experimental Setup and Results

To examine how porosity influences the propagation characteristics of guided waves and the generation of coda waves, several TC4 AM components were fabricated via SLM on an EOS M290 system. All specimens were fabricated using identical equipment and a single batch of powder to mitigate the influence of printing equipment variability and powder discrepancies on specimen properties. Table 1 outlines the chemical composition of the TC4 powder employed. The key printing parameters were set as follows: the laser spot size was 100 μm, the laser scanning velocity was 1900 mm/s, the layer thickness was 0.03 mm, and the hatching distance was 0.13 mm. In addition, the stripe scanning strategy was employed during printing, with a 67° rotation of the laser scanning direction between adjacent layers. By adjusting the laser power from 150 W to 350 W, AM components with varying porosity levels, which were designated as Specimen A through Specimen F, were fabricated. Each specimen was manufactured with final dimensions of 200 mm in length, 50 mm in width, and 3 mm in thickness. To mitigate deformation and residual stress in AM components, annealing heat treatment (800 °C for 2 h, furnace cooling) was conducted on the specimens prior to their cutting from the substrate.

**Figure 2:**
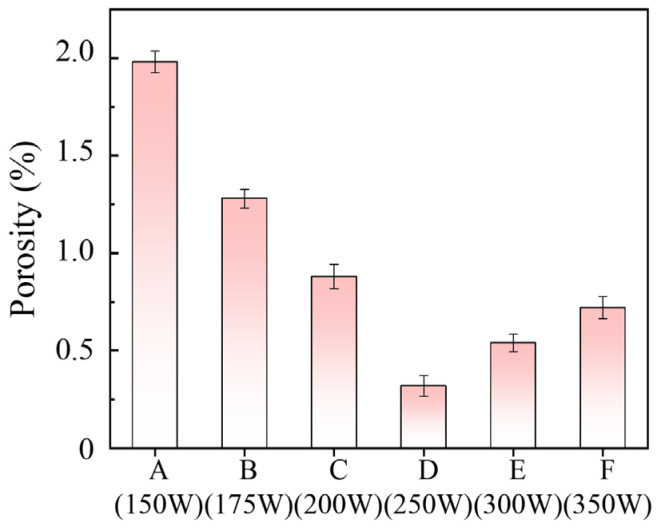
The relationship between laser power and the porosity of AM components.

**Figure 3:**
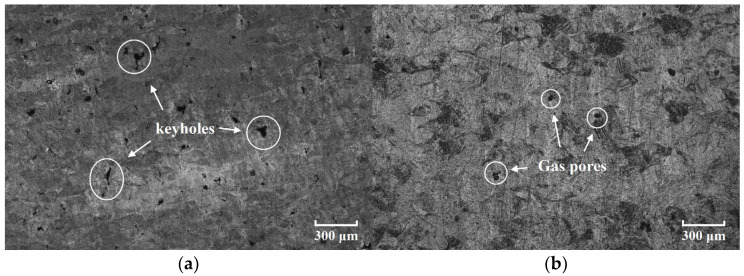
The metallographic images of AM components with different printing parameters: (**a**) Specimen A printing parameters and (**b**) Specimen F printing parameters.

The porosity of each AM component was determined using Archimedes’ principle, a widely recognized method for determining the relative density of AM components [32]. Prior to measurement, all specimens were first subjected to vacuum drying at 80 °C for 8 h to eliminate potential interference from residual moisture. For each specimen, ten independent density measurements were performed by fully immersing the specimen in water without contact with the container walls. The dense TC4 alloy exhibited a density of 4.43 g/cm^3^, and based on this reference value and these measurement results, the porosity and corresponding error bars of each specimen were calculated. As shown in Figure 2, the porosity of AM components exhibits a non-monotonic, V-shaped trend with increasing laser power, which initially decreases before reaching a minimum and then increases. This behavior arises from two distinct mechanisms. At low laser power, insufficient energy leads to incomplete melting of the powder, leading to the formation of keyholes [33]. Conversely, at excessively high laser power, the intense energy input causes violent melt pool dynamics, trapping protective gas and forming gas pores [34]. Taking the specimens fabricated with the printing parameters of Specimen A and Specimen F as examples, their metallographic plots are presented in Figure 3. It can be observed that the internal porosity of AM components exhibits significant differences under different printing parameters, and this result is highly consistent with the porosity measured by the Archimedes’ principle. In addition, the morphological characteristics of pores inside AM components also show distinct differences under different energy input conditions. It should be noted that although the specimens used for metallographic characterization were fabricated with exactly the same printing parameters as Specimen A and Specimen F, they were not subjected to annealing treatment. Both keyholes and gas pores affect guided wave propagation by inducing scattering, which in turn generates coda waves. Given that parameter optimization for new AM materials is often a lengthy and costly process, a suitable NDE method for high-precision porosity assessment is essential.

**Figure 4:**
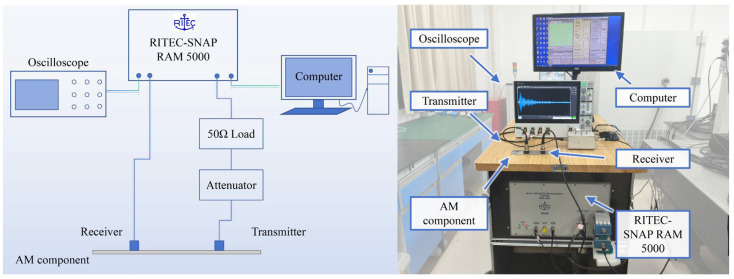
Experimental setup.

The experimental setup for assessing the porosity of AM components using ultrasonic guided waves is illustrated in Figure 4. Taking into account the wavelength, mode characteristics, and corresponding excitation method of ultrasonic guided waves, we have selected the A0 mode at 0.5 MHz as the primary wave. A RITEC-SNAP RAM 5000 system was used as the signal generator, and longitudinal wave transducers (Olympus A413S-SB center frequency: 0.5 MHz, Olympus, Tokyo, Japan) were employed for the excitation and reception of guided waves. The high-viscosity coupling agent (Olympus SWC-2, Olympus, Tokyo, Japan.) was employed to ensure acoustic coupling among the ultrasonic transducer and specimens. Furthermore, all assessed specimens were polished to guarantee uniformity in geometric dimensions and mitigate the interference of surface roughness on coda waves. During each assessment, the distance between the excitation and reception transducers was strictly controlled at 60 mm. The excitation signal is a 5-cysles Hanning window modulation signal, and the received signals were captured and displayed using a digital oscilloscope (Tekronix MSO44, Tektronix, Beaverton, OR, USA). To enhance the SNR, the received signals were amplified with a gain of 60 dB. Furthermore, each assessment signal represents the average of 128 independent data acquisitions. This approach effectively suppresses random noise and instantaneous coupling variability, thereby significantly enhancing the SNR and measurement repeatability.

**Figure 5:**
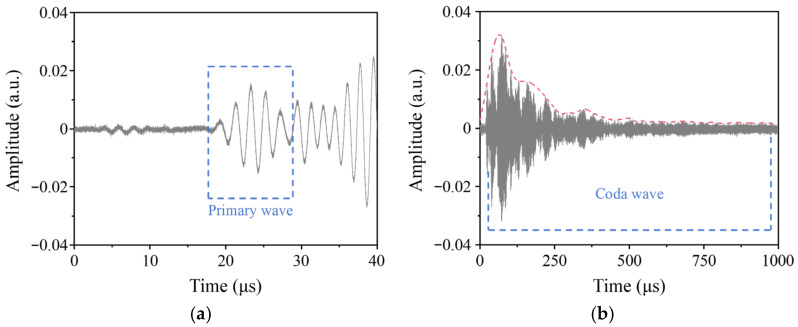
Typical ultrasonic guided wave received signals for porosity assessment in AM components: (**a**) the primary wave; (**b**) the coda wave.

Taking Specimen E as an example, Figure 5 illustrates typical assessment signals. The primary wave in Figure 5a yields a group velocity of 3157 m/s from TOF calculation, differing marginally by 0.76% from the theoretical value 3133 m/s. However, the primary wave is not suitable for porosity assessment, as the minute internal pores in AM components cause negligible perturbation to its propagation. Figure 5b displays the coda wave in the received assessment signal. The characteristics of this coda wave align with prior findings in coda wave analysis. With increasing TOF, the coda wave amplitude exhibits a valley-like trend, rising initially before gradually decaying. The initial amplitude increase is caused by the constructive interference of waves generated by internal scattering. However, with increasing TOF, the ultrasonic waves traverse longer paths, leading to greater energy attenuation from material damping and scattering, which ultimately dominates and causes the amplitude to decay.

**Figure 6:**
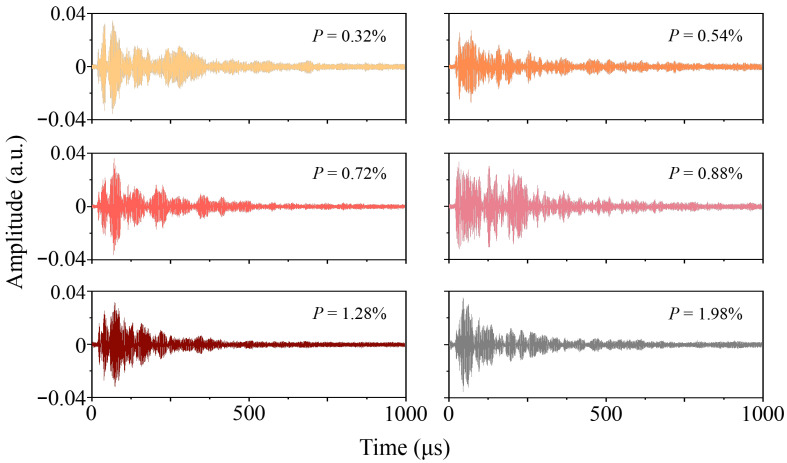
Ultrasonic coda wave assessment signals obtained from AM components with different porosity levels.

Figure 6 displays the coda wave assessment signals for AM specimens with varying porosity levels. The acquisition areas of these signals were not fixed, so as to ensure that the acquired signals could reflect the intrinsic porosity characteristics of each specimen rather than locally accidental structural features. The observable morphological changes in the coda wave indicate that it contains information relevant to porosity. This is because variations in internal pores alter the wavefield and propagation paths of ultrasonic guided waves. To analyze these signals, the waveform stretching method was employed. Due to the lowest porosity of Specimen D, the assessment signal of this specimen is used as the benchmark. The coda waves from other specimens were stretched or compressed, and then the correlation coefficient between each processed waveform and the reference was calculated. The maximum value of this coefficient corresponds to the stretching factor, ε, which represents the relative velocity change (*dv*/*v*) of the coda wave. The correlation coefficient *R_k_*(ε), which quantifies the similarity between the two coda waves, is defined as follows [31]:

(9) $$R_{k}(\varepsilon )=\frac{\int_{0}^{T}u^{\prime }(t)u(t)dt}{\sqrt{\int_{0}^{T}{u^{\prime }}^{2}(t)dt\cdot \int_{0}^{T}u^{2}(t)dt}},$$

where *u*(*t*) denotes the reference coda wave set, *u*′(*t*) is the coda wave from other assessed components after stretching or compression, and *T* is the duration of the entire coda wave. In addition, the relative velocity change is calculated as follows [31]:

(10) $$\left\{\begin{array}{c} t=\int_{l_{1}}^{l_{2}}\frac{1}{v}dl\\ t+t_{s}=\int_{l_{1}}^{l_{2}}\frac{1}{v+dv}dl=\int_{l_{1}}^{l_{2}}\left(\frac{1}{v}-\frac{dv}{v^{2}}\right)dl \end{array}\right.,$$

where *l*_1_ and *l*_2_ denote the starting and ending positions of the selected time window, and *t_s_* denotes the time delay. The subsequent solution of the equation is given by:

(11) $$\frac{t_{s}}{t}=-\frac{dv}{v}.$$

**Figure 7:**
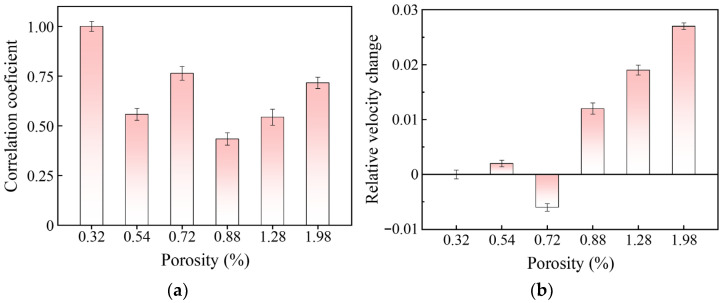
Relationship between porosity of AM components and characteristics of coda waves: (**a**) the correlation coefficient; (**b**) the relative velocity change.

The correlation coefficients and relative velocity changes in the assessment signals for AM components with different porosity levels were calculated using Equations (10) and (11), and the results are presented in Figure 7. To avoid interference from local structural features on the assessment results, multiple independent and distinct detection positions were selected on each specimen. As mentioned previously, only the detection position was adjusted for each assessment, while all other detection conditions were kept consistent. Multiple sets of coda wave signals were collected to ensure that the spatial diversity of porosity characteristics could be captured. Based on the data collected above, correlation coefficients, relative velocity changes, and corresponding error bars (the Standard Deviation, SD) were calculated. One-way Analysis of Variance (ANOVA) was performed separately on these two calculated parameters, and the outcomes revealed that both *p*-values are less than 0.001. This result indicates that the porosity of AM components exerts a highly statistically significant effect on the correlation coefficient and relative velocity change of coda waves. Although these two parameters are sensitive to changes in the coda wave, they exhibit no clear linear relationship with the porosity of the AM component. This complexity arises because the porosity in AM components is characterized by multi-dimensional micro-structural features, such as the number, size, and spatial distribution of pores. The mismatch between these complex input dimensions and the single-target feature (porosity) makes it difficult to establish a direct linear relationship using individual parameters. Consequently, accurately assessing porosity with traditional coda wave analysis methods remains challenging.

## 4. Porosity Evaluation by Integration of DL with Coda Waves

### 4.1. Proposed Coda-CNN-MAM Network

**Figure 8:**
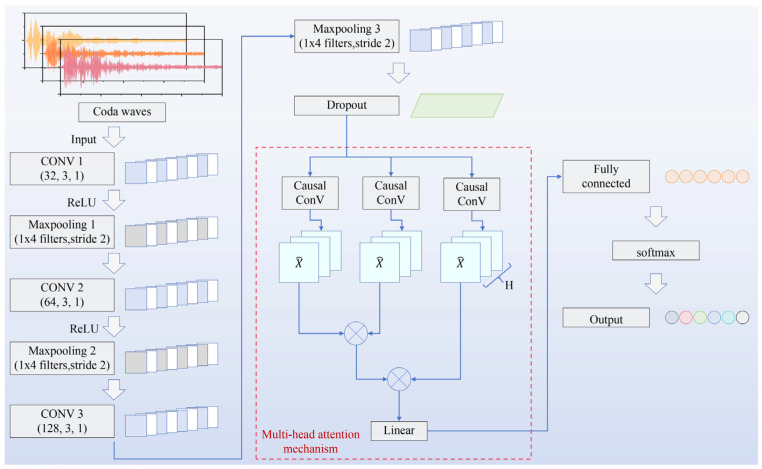
Schematic of the Coda-CNN-MAM network used for porosity assessment of AM components.

As demonstrated in the analysis results of the previous section, traditional coda wave analysis methods struggle to establish an effective mapping relationship with the porosity of AM components, thereby hindering the accurate evaluation of porosity. To address this limitation, we introduce the deep learning and propose a Coda-CNN-MAM network. Specifically, this network is designed to extract porosity-relevant features from coda wave signals, enabling precise porosity evaluation for AM components. The network architecture is illustrated in Figure 8. By leveraging the powerful feature extraction capability of CNN, the proposed model performs end-to-end learning and hierarchically extracts abstract features, from shallow to deep representations, directly from the raw coda wave signals [35]. CNN has been widely used in NDT. Junges et al. [36] combined Structural Health Monitoring (SHM) with DL to reduce the impact of Environmental and Operational Conditions (EOCs) on the performance of damage diagnosis methods, while mitigating the interference of temperature variations on ultrasonic guided wave-based SHM. Deng et al. [37] utilized CNN to analyze the ultrasonic evaluation signals of composite materials, and ultimately achieved accurate visualization of the location and characteristics of defects in composite material plates. The above research demonstrates the potential of CNN in processing ultrasonic assessment signals. However, unlike the processing of the primary wave signals in the aforementioned CNN applications, coda waves contain a large amount of physical information, including the ultrasonic scattered field, as well as the distribution characteristics of pores in the AM component. Such information typically exhibits complex long-range dependencies in the signal sequence. Nevertheless, the CNN is limited by the structural characteristics of its local receptive field, making it difficult to effectively capture such global contextual information, becoming a key bottleneck in its processing of coda wave signals [38]. Therefore, to effectively capture and integrate these deep-level features, we introduced a MAM module. The MAM deploys multiple independent attention heads in parallel, enabling the model to learn diverse dependencies within signals from different representation subspaces [39]. Each attention head independently calculates the correlation weights between different time positions, thereby capturing features dominated by different physical mechanisms, such as pore distribution in AM components and ultrasonic scattered field. This mechanism effectively enhances the modeling ability of long-range signal dependencies, thereby improving the feature resolution ability of the model in complex coda wave analysis and providing a more powerful decision-making algorithm for inverting material micro structures from ultrasonic coda waves.

The architecture of the proposed Coda-CNN-MAM network is illustrated in Figure 8. The CNN component mainly consists of three primary modules: convolutional layers, pooling layers, and fully connected layers. As the core module of the CNN, convolutional layers extract features from input data through convolutional kernels. The model incorporates three convolutional layers, containing 32, 64, and 128 kernels of size 1 × 3, respectively. Each convolutional layer is followed by a pooling layer with a window size of 1 × 4, adopting a max-pooling strategy to achieve dimensionality reduction of feature maps while retaining key salient features. The extracted features are subsequently integrated and transformed through fully connected layers. To enhance the network’s nonlinear modeling capability, all neurons use the Rectified Linear Unit (ReLU) as the activation function, ensuring the network can effectively capture the complex non-monotonic mapping relationship between input coda signals and porosity. Furthermore, a dropout layer is incorporated to mitigate overfitting, reduce the sensitivity of the network to small changes in the input data, and improve the network’s robustness. The core function of the Dropout layer is to break the co-adaptation relationships between neurons by randomly deactivating some neurons, thereby reducing the model’s over-reliance on local noise and idiosyncratic features in the training data (such as non-core features like accidental coupling noise at a specific detection position). This forces the model to learn more general and robust core patterns, ultimately enhancing its generalization ability and alleviating overfitting. As demonstrated by numerous studies [40,41], the introduction of this layer can effectively reduce the overfitting phenomenon.

To further boost the network’s capacity to model global correlations among features, the MAM is incorporated prior to the fully connected layer. The core essence of this mechanism lies in capturing the multivariate correlation relationships of input sequences in different feature subspaces [42]. This is achieved by computing multiple independent attention heads in parallel, and ultimately concatenating the outputs of all attention heads to form a more comprehensive global contextual representation. This representation can robustly capture global contextual information and serve as a powerful complement to the local features extracted by convolutional layers.

Specifically, the operation of MAM can be decomposed into four major steps: linear projection, head splitting, parallel scaled dot-product attention, and output concatenation [42]. Among these, linear projection involves applying three sets of independent learnable linear transformations to the input Query (Q), Key (K), and Value (V) vectors, respectively, mapping them from the original model dimension to the target dimension of attention heads. This not only completes dimensional adaptation for the subsequent head splitting but also injects differentiated feature representations through linear transformations. In the proposed Coda-CNN-MAM model, eight attention heads are adopted. The projected vectors were evenly split according to this number, and scaled dot-product attention was calculated independently for each split sub-vector to reveal the dependencies among multi-dimensional distinct features within different subspaces. Finally, the outputs of all attention heads were concatenated, and a final attention-based feature was obtained through a learnable linear transformation. The above operations not only enhance the model’s parallel computing capability but also enable the model to capture key features in the input information from different perspectives and dimensions, significantly improving the model’s feature expression capability and learning efficiency.

Herein, the distinct features mentioned above refer to those in coda wave signals with significant discriminability and strong correlation with porosity. Pores of different microstructural features uniquely alter the propagation paths and characteristics of coda waves, and this specific interaction allows coda waves to effectively assess AM component porosity. However, conventional analysis methods cannot accurately extract such porosity-correlated coda wave features, which is the core reason for introducing DL in this study.

The configuration and hyperparameters of the proposed Coda-CNN-MAM network architecture plays a pivotal role in enhancing the performance of DL networks. In this study, the Adam (Adaptive Moment Estimation) optimizer was employed to iteratively adjust and identify an optimal set of hyperparameters for the Coda-CNN-MAM network. The learning rate was initialized at 0.01 and further refined using the gradient descent algorithm. To evaluate model performance during training, the cross-entropy loss was adopted as the loss metrics. To ensure sufficient data availability, a total of 600 assessment signals were collected, with 100 signals acquired from each specimen. The distance between the transmitter and receiver was consistently maintained at 60 mm, while the assessment region was not kept constant during detection so as to capture the spatial diversity of porosity characteristics. Prior to input into the Coda-CNN-MAM network, the primary waves in the assessment signals were eliminated. The complete dataset was randomly split into training and testing sets at a ratio of 7:3. The training set was used for model estimation and architectural optimization, and the testing set was reserved for evaluating the final network performance. To establish an effective nonlinear mapping between the processed coda signals and porosity, parameters including convolutional kernels, weights, and biases were iteratively optimized with the objective of minimizing the cross-entropy loss, thereby enhancing prediction accuracy. All computations were performed on a workstation equipped with 512 GB of RAM, an Intel Xeon Gold 6230 R CPU, and an NVIDIA GeForce RTX 3080 Ti GPU (NVIDIA, Santa Clara, CA, USA).

### 4.2. Prediction Results and Discussions

**Figure 9:**
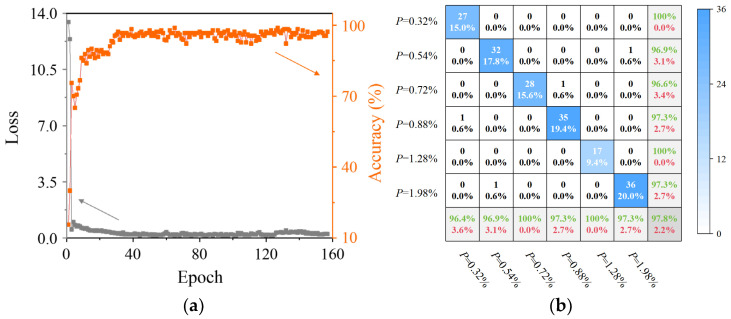
The training process and results of the proposed Coda-CNN-MAM network: (**a**) curve of loss and accuracy vs. epoch; (**b**) classification confusion matrix under different porosity levels.

The processed coda wave assessment signals were trained using the proposed Coda-CNN-MAM network. Figure 9 illustrates the loss and classification accuracy on the test set throughout the training process. The loss on the test set demonstrates a steady decline and eventually stabilizes, indicating good model convergence and effective mitigation of overfitting or underfitting. Concurrently, the test accuracy consistently improves and plateaus at a high level, affirming the network’s robust capability in capturing and learning porosity-related features from the coda wave signals. The classification accuracy of the network reached 97.8%, and the final classification result is shown in Figure 9b, demonstrating its ability to distinguish the coda wave signals of AM components with different porosity levels. This classification confusion matrix visually represents the classification performance of the model across six porosity levels. The rows correspond to the ground-truth porosity labels of the specimens, while the columns indicate the predicted porosity labels by the model. Each cell displays the count of signals classified into the corresponding category and their respective percentage, with the numerical values and color intensity collectively reflecting the distribution of classification results. The color bar on the right, represented by a gradient of blue, indicates the number of signals in each cell, with darker shades indicating a greater number of signals in that category. The misclassification rate remained remarkably low across all specimens, peaking at only 3.6%. These high-precision classification outcomes indicate that the proposed network can effectively excavate the deep-seated features related to component porosity in coda wave signals, enabling efficient and accurate porosity assessment of AM components. In addition, as assessment was performed at multiple distinct locations across each specimen, these results further demonstrate that the model learned porosity-related features consistent across the specimen, rather than random coupling noise or systematic perturbations. The above results further confirm that the model exhibits excellent generalization ability. Varying the detection positions allows for capturing the spatial diversity of porosity characteristics, preventing the model from merely fitting the signal features of local positions. In terms of network structure design, the introduction of the dropout layer reduces sensitivity to minor variations in input and mitigates the risk of overfitting. More crucially, the integration of the MAM compensates for CNN’s insufficiency in global feature extraction, enabling it to capture the global contextual features related to porosity in coda wave signals. These measures have effectively improved the model’s generalization ability, ensuring that the model learns universal features associated with porosity rather than random coupling noise or systematic perturbations.

To more comprehensively and robustly evaluate the model performance, we conducted stratified k-fold cross-validation. Specifically, the dataset corresponding to each porosity category were randomly partitioned into 5 equal folds, and each fold was sequentially used as the test set while the remaining folds served as the training set in a cyclic manner. The cross-validation results show that the model achieved an average classification accuracy of 97.3% ± 0.8% across the 5 test folds, with consistent recognition performance observed across all categories. These findings further confirm that the proposed Coda-CNN-MAM network exhibits excellent classification robustness and generalization capability under varying data distributions.

**Figure 10:**
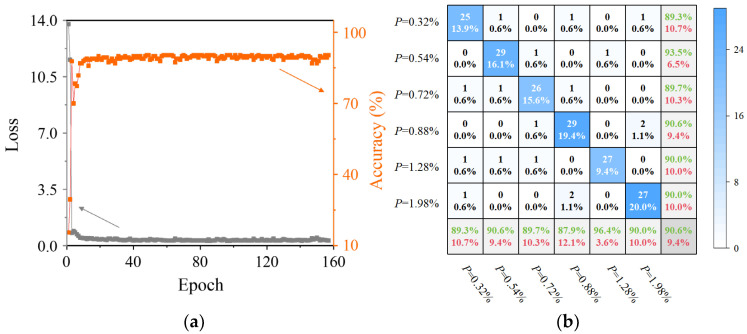
The training process and results of the CNN trained on the coda wave signals: (**a**) curve of loss and accuracy vs. epoch; (**b**) classification confusion matrix under different porosity levels.

To validate the effectiveness of the proposed Coda-CNN-MAM network, comparative experiments were conducted: one employing a standalone CNN for the coda wave signals, and another training the proposed network only using the primary wave signals. The performance of the standalone CNN trained using the coda wave signals was first evaluated. Figure 10a shows the loss and classification accuracy during its training process, while the final classification results are presented in Figure 10b. The classification results indicate that while the standalone CNN achieves some level of classification accuracy (90.6%), its performance is substantially inferior to the proposed Coda-CNN-MAM network (97.8%), representing a notable decrease of 7.2%. Furthermore, the CNN exhibited a significantly higher misclassification rate, exceeding 12.1% in certain porosity levels, which stands in sharp contrast to the maximum rate of 3.6% for the proposed network. The performance gap primarily stems from the standalone CNN’s reliance on convolution operations, which excel at extracting local features but struggle to capture the deep global correlations within the coda wave signals that are indicative of AM component porosity. In contrast, the Coda-CNN-MAM network overcomes this limitation by integrating the MAM, enabling a more comprehensive and accurate feature extraction process. The advantage of MAM lies in its ability to effectively capture the long-range contextual correlation of coda wave signals, which is difficult to achieve with the traditional CNN. In addition, its multi-head design avoids over-reliance on single-scale feature correlation, thereby enhancing the robustness of porosity feature extraction. On the downside, MAM marginally increases the computational cost of the network, resulting in a 9.56% increase in training time. Coda wave signals generated via multiple scattering and reflection within AM components exhibit strong non-local correlations, such that porosity-related features are not confined to a specific segment of the coda wave signal. Consequently, MAM delivers superior performance in this context. The comparative results validate the effectiveness and marked superiority of the proposed DL network for the porosity assessment of AM components.

**Figure 11:**
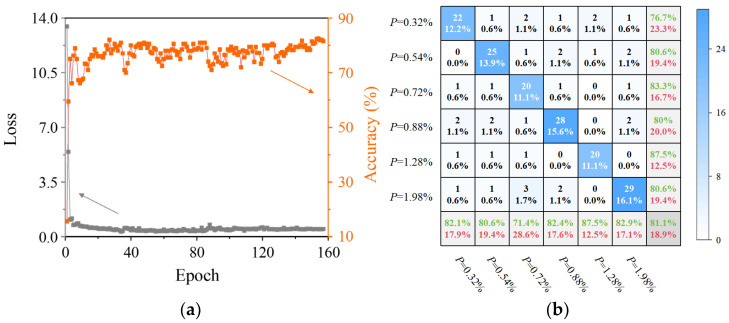
The training process and results of the proposed Coda-CNN-MAM network trained exclusively on the primary wave signals: (**a**) curve of loss and accuracy vs. epoch; (**b**) classification confusion matrix under different porosity levels.

Further analysis was conducted on the performance of the proposed Coda-CNN-MAM network trained exclusively on the primary wave signals. The loss and classification accuracy curves of the test set during network training are illustrated in Figure 11a, and alongside those of the aforementioned models, these results collectively indicate that the model in this study is not significantly affected by overfitting. The losses of both the training set and the test set decreased steadily and converged to a stable level, with no typical characteristics of overfitting such as a rebound in the test set loss or a significant widening of the loss gap between the training set and the test set. A series of measures, ranging from data collection to the targeted design of the model structure, have effectively mitigated the risk of significant overfitting of the model with a limited dataset. The final classification results are presented in Figure 11b, which indicates that the classification accuracy of the Coda-CNN-MAM network decreased significantly to 81.1% when relying solely on primary wave signals. This represents a notable drop of 16.7% compared to the 97.8% accuracy achieved when trained on coda wave signals. Meanwhile, the misclassification rate of specimens increased substantially, with the rate reaching 28.6% in certain porosity levels, which far exceeded the maximum 3.6% rate observed in coda wave signal training results. This discrepancy arises from the fundamental differences in the wave propagation characteristics. The primary wave propagates over a shorter path within the AM component, thereby capturing only limited information and making it difficult to reveal deep features related to porosity. In contrast, the coda wave undergoes multiple scattering and reflections, integrating structural information from various locations. This process imbues the coda wave with richer porosity-related features, making it more compatible with the feature extraction logic of the Coda-CNN-MAM model.

Based on the above analysis, the performance of the Coda-CNN-MAM model proposed in this study is attributed to the synergistic effect of physical mechanisms and network architecture. The physical characteristics of ultrasonic coda waves form the core foundation underpinning the model’s performance. Propagating through AM components via multiple scattering and reflection, coda waves can fully interact with pores and thus carry multi-dimensional microstructural features associated with porosity. This physical characteristic endows the coda wave signals with relatively strong feature discriminability, which also serves as an important prerequisite for the model to accurately extract porosity-related features. In addition, tailored to this input characteristic of coda wave signals, an adaptive network architecture was designed, whose core advantage lies in the synergistic effect formed by the local feature extraction capability of the CNN and the global contextual modeling capability of the MAM. Collectively, these findings validate the effectiveness of the Coda-CNN-MAM architecture, laying a robust methodological foundation for high-precision non-destructive porosity assessment of AM components.

### 4.3. Limitations and Future Work

Although the proposed Coda-CNN-MAM network demonstrates high accuracy in porosity assessment for AM components, several limitations should be acknowledged, which also indicate directions for future research.

First, the current study was confined to TC4 alloy and the specimens fabricated by SLM. Different AM materials (e.g., AlSi10Mg, 316L stainless steel) and AM processes (e.g., Electron Beam Melting, EBM) induce distinct pore characteristics and ultrasonic wave propagation characteristics, which could affect the model’s generalization. Future work will validate the model on diverse materials and processes, and adopt transfer learning to fine-tune the pre-trained network with small-sample data, thereby reducing the demand for large-scale labeled datasets.

Second, the current study was conducted under controlled laboratory conditions. In industrial scenarios, temperature fluctuations, surface roughness variations, and dynamic coupling stability may distort coda wave signals. These factors were not fully considered in the current experiments, limiting the model’s direct applicability in on-site monitoring. Subsequent research will integrate environmental compensation mechanisms to enhance the model’s robustness in complex industrial environments.

## 5. Conclusions

In this paper, we proposed a new NDE method for assessing porosity in AM components using a DL-assisted ultrasonic technique. Recognizing the high sensitivity of ultrasonic coda waves to micro internal alterations, we utilized this ability to assess the porosity of AM components. Since the porosity in AM components is characterized by multi-dimensional micro-structural features, the traditional coda wave analysis methods struggle to establish a linear relationship between coda wave and porosity. To address this, we introduced a Coda-CNN-MAM network, which capitalizes on the capability of DL in feature extraction and decoupling of complex nonlinear mappings. The network effectively combines the rich porosity-related information carried by coda waves, the local feature extraction ability of CNN, and the global contextual modeling capacity of the MAM module, achieving high-precision porosity assessment with an accuracy of up to 97.8%. By comparing separately with the assessment results of the CNN trained only on coda wave signals and the proposed network trained only on the primary wave signals, the significant effectiveness and prominent advantages of the proposed network in the porosity assessment of AM components are verified. This work establishes an NDE framework that integrates ultrasonic coda waves with advanced DL architectures, offering an accurate and efficient solution for porosity assessment in AM components.

## Figures and Tables

**Table 1:** Chemical composition of TC4 particles (wt. %).

Al	V	Fe	O	C	N	Ti
6.32	4.02	0.21	0.11	0.05	0.03	Remain

## Data Availability

Data are contained within the article.
